# Modulating the immunodominance hierarchy of immunoglobulin germline-encoded structural motifs targeting the influenza hemagglutinin stem

**DOI:** 10.1016/j.celrep.2024.114990

**Published:** 2024-11-22

**Authors:** Sila Ataca, Maya Sangesland, Rebeca de Paiva Fróes Rocha, Alba Torrents de la Peña, Larance Ronsard, Seyhan Boyoglu-Barnum, Rebecca A. Gillespie, Yaroslav Tsybovsky, Tyler Stephens, Syed M. Moin, Julia Lederhofer, Adrian Creanga, Sarah F. Andrews, Ralston M. Barnes, Daniel Rohrer, Nils Lonberg, Barney S. Graham, Andrew B. Ward, Daniel Lingwood, Masaru Kanekiyo

**Affiliations:** 1 Vaccine Research Center, National Institute of Allergy and Infectious Diseases, National Institutes of Health, Bethesda, MD 20892, USA; 2 Ragon Institute of MGH, MIT and Harvard, Cambridge, MA 02139, USA; 3 The Scripps Research Institute, La Jolla, CA 92037, USA; 4 Vaccine Research Center Electron Microscopy Unit, Cancer Research Technology Program, Frederick National Laboratory for Cancer Research, Frederick, MD 20701, USA; 5 Bristol-Myers Squibb, Redwood City, CA 94063, USA

**Keywords:** influenza virus, broadly neutralizing antibody, bnAb, hemagglutinin, germline-targeting, HA stem, nanoparticle, B cell, IGHV1-69, IGHD3-9

## Abstract

Antibodies targeting epitopes through germline-encoded motifs can be found in different individuals. While these public antibodies are often beneficial, they also pose hurdles for subdominant antibodies to emerge. Here, we use transgenic mice that reproduce the human IGHV1-69^∗^01 germline-encoded antibody response to the conserved stem epitope on group 1 hemagglutinin (HA) of influenza A virus to show that this germline-endowed response can be overridden by a subdominant yet cross-group reactive public antibody response. Immunization with a non-cognate group 2 HA stem enriched B cells harboring the IGHD3-9 gene, thereby switching from IGHV1-69- to IGHD3-9-encoded motif-dependent epitope recognition. These IGHD3-9 antibodies bound, neutralized, and conferred cross-group protection in mice against influenza A viruses. A cryoelectron microscopy (cryo-EM) structure of an IGHD3-9 antibody resembled the human broadly neutralizing antibody FI6v3, which uses IGHD3-9. Together, our findings offer insights into vaccine regimens that engage an immunoglobulin repertoire with broader cross-reactivity to influenza A viruses.

## Highlights

• Group 2 stem priming enriched cross-group reactive B cells upon group 1 stem boost
• Cross-group reactive antibodies use IGHD3-9- instead of the IGHV1-69-encoded motif
• IGHV1-69 + IGHD3-9 antibodies neutralize and confer cross-group protection in mice
• IGHD3-9-encoded motif facilitates HA stem recognition analogous to human bnAb FI6v3

## Graphical abstract

**Figure:**
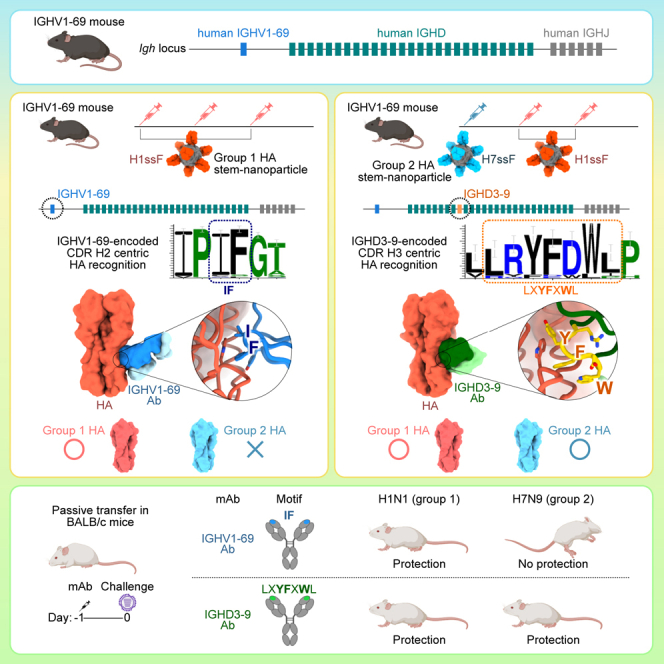

## Teaser

Ataca et al. describe a pathway to generate cross-reactive B cells against influenza hemagglutinin by engaging a subdominant immunoglobulin germline-encoded motif through prime-boost vaccination in IGHV1-69 mice. This study provides a blueprint for the vaccine elicitation of broader B cell responses in the face of predominant B cells with narrower cross-reactivity.

## Introduction

Influenza A and B viruses cause seasonal epidemics and hundreds of thousands of hospitalizations and deaths every year.^1^ Influenza A viruses (IAVs) have also been responsible for four major global pandemics in the last 110 years. IAVs have two surface glycoproteins, hemagglutinin (HA) and neuraminidase (NA), that are divided into 18 and 11 subtypes, respectively. HA subtypes can be clustered into two groups based on their sequences. The IAVs that currently circulate in human populations belong to H1N1 and H3N2, which possess group 1 HA (H1) and group 2 HA (H3), respectively. Thus, current seasonal vaccines contain these IAVs as well as one or two lineages of influenza B viruses to provide coverage. While the current vaccines are capable of eliciting antibodies directed toward the hypervariable globular head domain of HA, they often result in suboptimal performance due to antigenic mismatch between the vaccine and circulating viral strains. Indeed, seasonal vaccines typically show vaccine effectiveness against symptomatic disease ranging from 10% to 60% in a given season.^2^ In addition, avian influenza viruses with pandemic potential, such as H5N1 and H7N9, cause sporadic human infections worldwide and pose serious threats to public health. There is a clear need for a universal influenza vaccine that can elicit broader protective immunity. Therefore, universal vaccine efforts aim to elicit broadly neutralizing antibodies (bnAbs) that target conserved sites of vulnerability such as the stem region of HA.^3^^,^^4^^,^^5^^,^^6^^,^^7^

In humans, HA-stem-directed bnAbs are often derived from public clonotypes or multidonor classes, featuring IGHV gene segments such as IGHV6-1^8^^,^^9^ and IGHV1-18^9^^,^^10^ with highly similar complementarity-determining region (CDR) H3 sequences, or public lineages, featuring the same immunoglobulin (Ig) gene usage with germline-encoded structural motifs to engage epitopic residues on the stem such as IGHV1-69,^11^^,^^12^^,^^13^^,^^14^ IGHV3-23,^15^^,^^16^ and IGHV3-30.^17^^,^^18^ Among these, the IGHV1-69 lineage is unique, as certain alleles of IGHV1-69 encode a pair of hydrophobic residues at the tip of the heavy-chain CDR 2 (CDR H2) loop, which engages the conserved hydrophobic groove surrounding the W21_HA2_ of the HA stem, serving as a germline-encoded pre-configured pattern recognition motif for the group 1 HA stem. With only a few mutations, these IGHV1-69 antibodies achieve high-affinity, intra-group cross-reactivity, making them an attractive lineage for vaccine elicitation of broad protection against group 1 IAVs.^12^^,^^19^ Upon primary exposure to non-circulating group 1 HA subtypes such as pandemic H1, H5, and H2, IGHV1-69 dominates antibody responses to the stem^20^^,^^21^^,^^22^^,^^23^^,^^24^^,^^25^; however, the differences in glycosylation between group 1 and group 2 HA stems render most IGHV1-69 antibodies incapable of binding the group 2 HA stem.^26^^,^^27^^,^^28^

Previously, we demonstrated that the stereotypic IGHV1-69 antibody responses could be reproduced in a transgenic IGHV1-69 mouse, which carries the complete human IGHD and IGHJ genes, allowing for the generation of a diverse, human-like CDR H3 repertoire.^29^^,^^30^^,^^31^ Monoclonal antibodies (mAbs) isolated from mice immunized with the H1 stabilized stem ferritin nanoparticle (H1ssF) possess the same immunogenetic characteristics as human IGHV1-69 antibodies to the group 1 stem.^29^ Thus, the IGHV1-69 mice allow us to not only study canonical HA-stem-directed antibodies but also test hypotheses that would otherwise not be possible to address except in humans, such as the elicitation of non-canonical subdominant antibodies in the face of germline-encoded predominant antibodies.

In this study, we show that under certain immunization conditions, the predominant IGHV1-69 germline-encoded CDR H2-dependent response can be replaced by a CDR H3-centric mode of recognition to accommodate the binding and neutralization of group 2 HAs that are otherwise incompatible with the canonical CDR H2-centric recognition. Antibodies adopting this alternative mode of recognition use the RYFDW motif, which is encoded by IGHD3-9 with a particular reading frame (RF) to target the same hydrophobic groove on the HA stem. Intriguingly, this IGHD3-9 motif is also found in stem-directed human bnAbs such as FI6v3,^10^^,^^32^^,^^33^ suggesting that IGHD3-9-centric antibodies can also be elicited in humans. This IGHD3-9 motif serves as a pre-configured determinant for the conserved stem of group 1 and group 2 HAs and can override the predominant antibody responses with narrower specificity under certain circumstances.

## Results

### Group 2 HA stem immunization alters the immune response in IGHV1-69 mice

**Figure 1:**
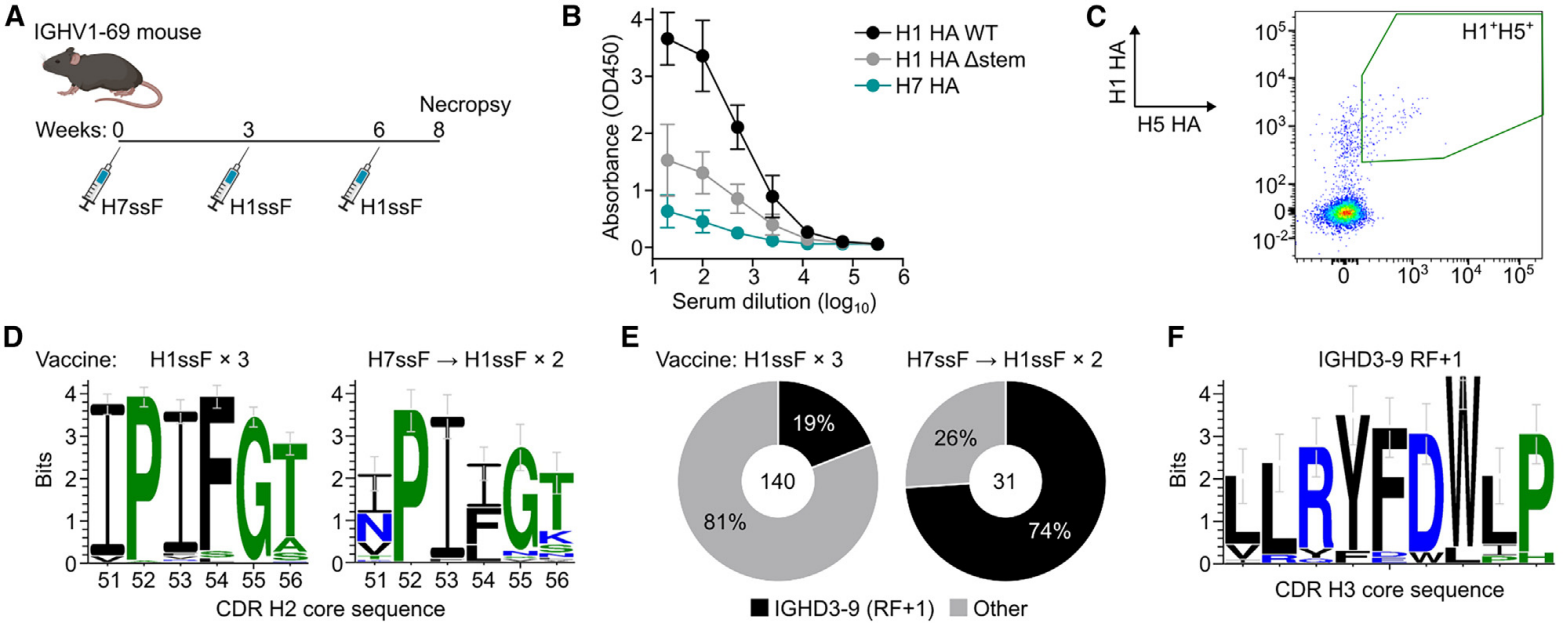
Characterization of immune responses in IGHV1-69 mice immunized with group 2 HA stem followed by group 1 HA stem (A) Immunization scheme of IGHV1-69 mice. Mice (*n* = 6) were primed (week 0) with 15 μg of H7ssF followed by two immunizations with H1ssF at weeks 3 and 6. Mice were bled at week 8. (B) Specificity of HA-binding serum antibody measured by ELISA. Mean and SEM are shown for each ELISA curve (*n* = 6). H1 HA WT, wild-type H1 HA of A/New Caledonia/20/1999; H1 HA Δstem, H1 HA with substitutions in the central stem epitope that knock out antibodies targeting the central stem; H7 HA, wild-type H7 HA of A/Anhui/1/2013. (C) Flow cytometry plot of H1^+^H5^+^ cross-reactive HA-specific B cell population within spleen. Complete gating scheme can be found in Figure S1. (D) Comparison of the CDR H2 amino acid sequences among H1^+^H5^+^ BCRs in IGHV1-69 mice immunized with homologous group 1 (H1ssF × 3, left) and group 2 followed by group 1 stem (H7ssF → H1ssF × 2, right). (E) Comparison of frequency of different reading frames (RFs) of IGHD3-9 gene within H1^+^H5^+^ BCRs in IGHV1-69 mice immunized with homologous group 1 (H1ssF × 3, left) and group 2 followed by group 1 stem (H7ssF → H1ssF × 2, right). (F) CDR H3 core amino acid sequences within H1^+^H5^+^ BCRs in IGHV1-69 mice immunized with group 2 followed by group 1 stem (H7ssF → H1ssF × 2). See also Figures S1 and S2.

Most IGHV1-69 antibodies isolated from humans or IGHV1-69 mice to date are predominantly group 1 HA specific. As such, we hypothesized that the IGHV1-69 germline-encoded CDR H2 motif is incompatible with the group 2 HA stem structure and therefore would not be responsive to immunization with the group 2 HA stem. To determine if priming with group 2 HA stem immunogens can elicit antibody responses with different specificity, sera from IGHV1-69 mice primed with the group 2 H7ssF^7^ followed by two boosts of group 1 H1-based H1ssF were characterized (Figure 1A). The immunization regimen elicited a strong serum antibody response targeting H1 HA, whereas the elicitation of antibodies to H7 HA was comparably weaker. Analogous to animals immunized three times with H1ssF,^29^ serum antibody binding to the H1 HA in the H7-primed mice was reduced when access to the central stem epitope was obstructed by I45R/T49R mutations (H1 Δstem HA),^12^^,^^34^ compared to an intact H1 HA (wild-type [WT] H1 HA), suggesting that the induction of central stem-specific antibodies was maintained when animals were primed with a group 2 stem immunogen (Figure 1B).

We next sought to characterize HA-specific B cells elicited by the H7ssF-primed and H1ssF-boosted IGHV1-69 mice by flow cytometry. We sorted B cells with cross-reactivity to H1 and H5 HAs (H1^+^H5^+^), which have been previously used to isolate stem-specific B cell receptors (BCRs) (Figures 1C and S1).^29^ BCRs of H1^+^H5^+^ B cells were sequenced and analyzed. Surprisingly, the majority of recovered heavy-chain sequences did not possess the hallmark phenylalanine at position 54 within the CDR H2 and instead encoded isoleucine at that position (Figure 1D). This F54I substitution is not observed in B cells isolated from the IGHV1-69 mice immunized with H1ssF alone (Figure 1D). Moreover, IGHD3-9 gene usage in the H7ssF-primed animals was substantially higher at 74% than in animals immunized only with H1ssF (19%), suggesting the positive selection of the IGHD3-9 gene among the H1^+^H5^+^ B cells through priming with a group 2 (H7ssF) immunogen (Figure 1E). The IGHD3-9 gene of these H1^+^H5^+^ B cells disproportionately used RF+1, which encodes the core RYFDW motif (Figures 1F and S2). In humans, the predominant RF for IGHD3-9 is RF+3, which is preferentially used over RF+1.^35^ Interestingly, both RF+1 and RF+2 were previously shown to associate with the recognition of the central HA stem epitope through encoded hydrophobic residues in humans.^9^^,^^10^^,^^32^^,^^33^ All IGHD3-9 sequences isolated in this study are paired with IGHJ3. This is in line with the observation that IGHD3-9 and IGHJ3 exhibit preferential pairing in the human repertoire.^36^

### Identification of IGHV1-69 + IGHD3-9 mAbs that are broadly cross-reactive and neutralizing

**Figure 2:**
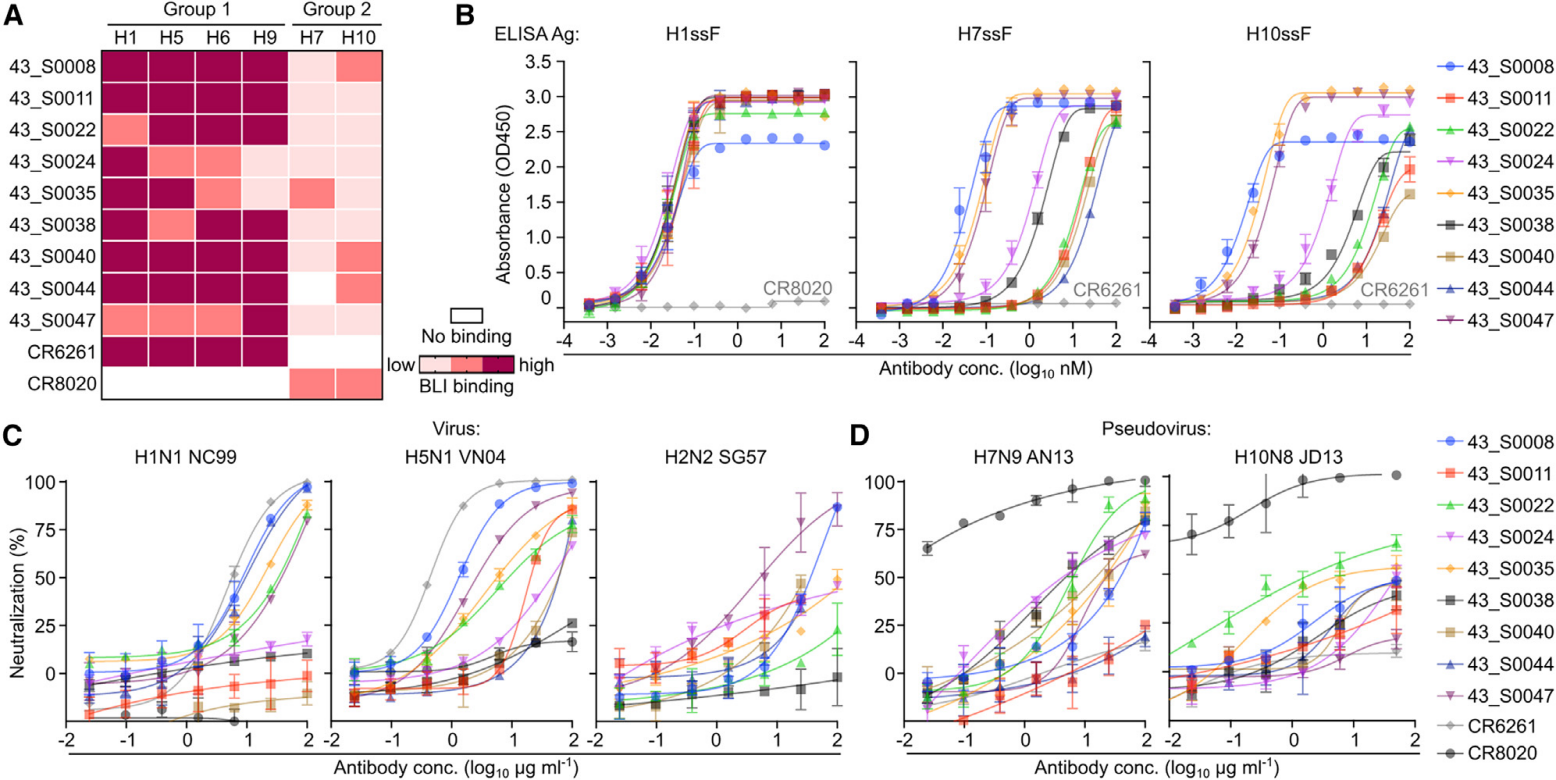
HA-binding and virus neutralization profiles of IGHV1-69 + IGHD3-9 mAbs (A) HA-binding profiles of the 9 cross-reactive IGHV1-69 + IGHD3-9 mAbs against group 1 and group 2 HAs by biolayer interferometry (BLI). White indicates no binding (<0.1 nm shift in BLI signal). Pink, salmon, and cayenne indicate low (0.1–0.5 nm shift), medium (0.5–1.0 nm shift), and high binding (1.0–1.5 nm shift), respectively. (B) HA-stem-binding profiles of the 9 cross-reactive IGHV1-69 + IGHD3-9 mAbs against group 1 and group 2 HA stems by ELISA. HA stabilized stem ferritin nanoparticles (ssFs) of H1, H7, and H10 were used as antigens. (C and D) Neutralizing activity of the 9 cross-reactive IGHV1-69 + IGHD3-9 mAbs against group 1 and group 2 viruses. Neutralization activity against H1N1, H5N1, and H2N2 was measured by a reporter-based microneutralization assay (C). Neutralization activity against H7N9 and H10N8 was measured by a pseudotyped lentivirus neutralization assay (D). Group 1 HA-stem-specific (CR6261) and group 2 HA-stem-specific (CR8020) mAbs were used as controls in binding and neutralization assays. See also Table S1.

To characterize the HA binding properties, the IGHV1-69 + IGHD3-9 mAbs were evaluated for their binding to various group 1 and group 2 HA subtypes by ELISA and biolayer interferometry (BLI). Consistent with the sorting profile, all IGHV1-69 + IGHD3-9 mAbs bound to H1 and H5 HAs (Table S1). Surprisingly, 9 out of 31 IGHV1-69 + IGHD3-9 mAbs showed cross-group reactivity despite being derived from the group 1-biased IGHV1-69^∗^01 allele (Figure 2A). These nine mAbs bound to H1, H5, H6, and H9 HAs from group 1 but also H7 and H10 HAs from group 2. Of note, none of these mAbs had detectable binding to H3 HAs. All nine mAbs bound the strongest to H1ssF, and binding to H7ssF and H10ssF appeared more heterogeneous (Figure 2B). To test the functionality of IGHV1-69 + IGHD3-9 mAbs, we assessed their neutralizing activity against H1N1, H2N2, and H5N1 viruses in the reporter-based microneutralization assays.^37^ Five out of nine tested mAbs neutralized homologous H1N1 (A/New Caledonia/20/1999 [NC99]) virus with either a higher or comparable half-maximal inhibitory concentration (IC_50_) to that of human IGHV1-69 mAb CR6261. We also detected neutralizing activity of our mAbs (8 of 9) against the H5N1 (A/Vietnam/1203/2004 [VN04]) virus, although they were not as potent as CR6261. Two mAbs also neutralized the H2N2 (A/Singapore/1/1957 [SG57]) virus (Figure 2C). In addition, we tested the mAbs against H7N9 (A/Anhui/1/2013 [AN13]) and H10N8 (A/Jiangxi-Donghu/346/2013 [JD13]) viruses using highly sensitive lentiviral pseudotype neutralization assays^38^^,^^39^ in which weaker HA-stem-directed neutralizing activity can be detected. We found that seven out of the nine mAbs exhibited neutralizing activity against H7N9, with weaker activity against H10N8 pseudoviruses (Figure 2D). These results show that IGHV1-69 + IGHD3-9 mAbs elicited in IGHV1-69 mice via group 2 HA stem priming can be cross-group reactive and neutralize viruses harboring group 1 and group 2 HAs.

### Structural characterization of cross-neutralizing antibody 43_S0008 to H1 HA

**Figure 3:**
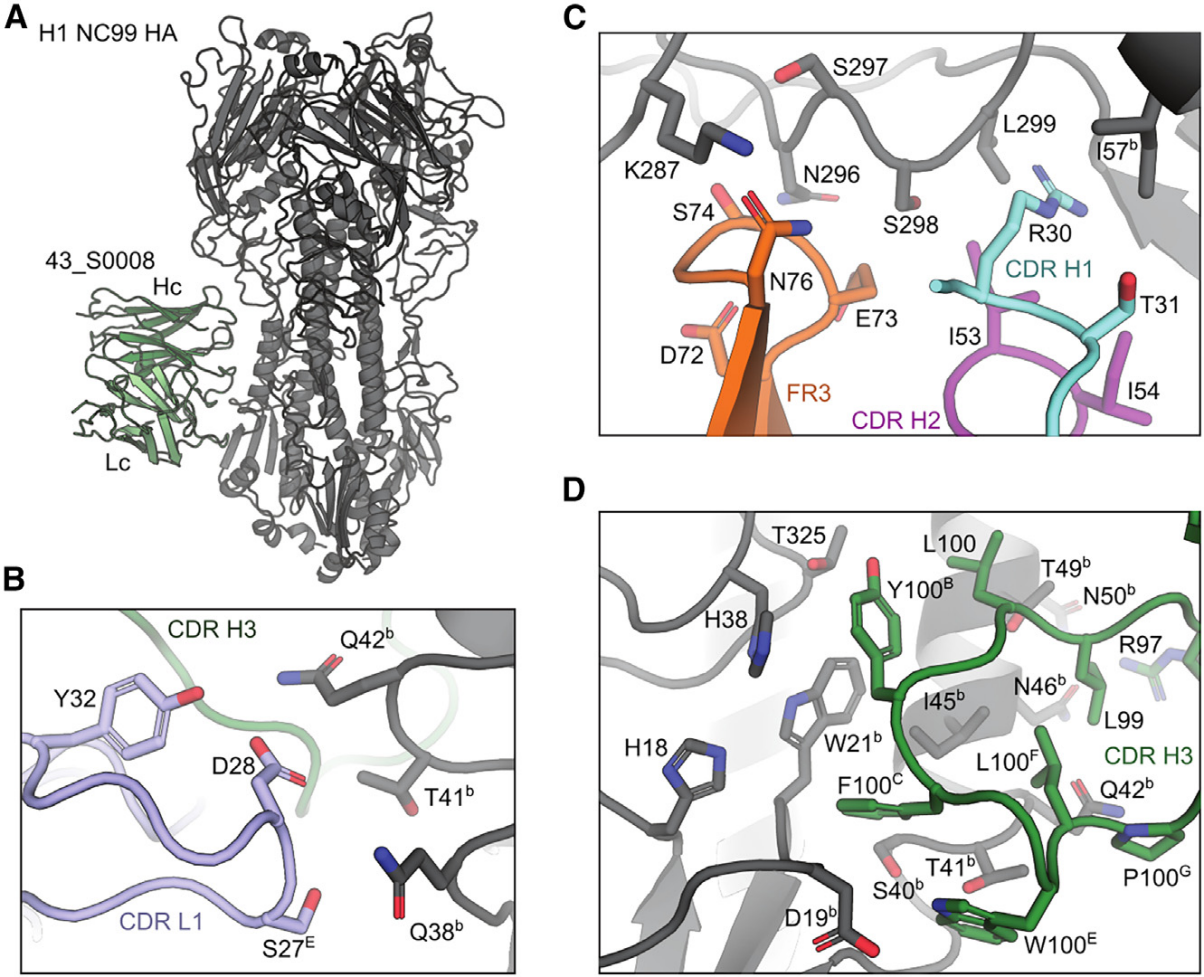
Cryo-EM structure of IGHV1-69 + IGHD3-9 43_S0008 Fab in complex with HA (A) Overall cryo-EM structure of Fab 43_S0008 in complex with HA. HA trimer is shown in gray and Fab 43_S0008 in green. (B–D) Detailed amino acid interactions of the HA trimer with Fab 43_S0008 light-chain CDR L1 and CDR H3 (B); FR3, CDR H1, and CDR H2 (C); and CDR H3 (D) are depicted in different colors according to the different Fab regions. See also Tables S2 and S3 and Figures S3 and S4.

To gain a comprehensive understanding of the interaction between the IGHV1-69 + IGHD3-9 mAb and HA, we solved the structures of a representative IGHV1-69 + IGHD3-9 mAb, 43_S0008, in complex with HA using cryoelectron microscopy (cryo-EM). The antibody was complexed with the HA trimer of influenza H1N1 NC99, and its structure was solved to 3.09 Å resolution (Figures 3A and S3; Table S2). We find that mAb 43_S0008 interacts with a highly conserved hydrophobic groove at the HA1/HA2 interface in the HA stem. The interaction of Fab 43_S0008 with H1 involves key interacting residues derived from CDR 2, CDR 3, and framework region (FR) 3 of the heavy chain with minimal light chain contacts (Figures 3B–3D; Table S3).

Similar to other IGHD3-9 mAbs that encode the LXYFXWL motif in the CDR H3 to recognize HA,^32^^,^^33^ the majority of contacts between Fab 43_S0008 and HA occur within residues L100, Y100^B^, F100^C^, W100^E^, L100^F^, and P100^G^ of the antibody (Figure 3D; Table S3). Of note, the CDR H3 aspartic acid residue at position 100^D^ is not part of the paratope, and we confirmed that the D100^D^A substitution, as demonstrated in our mutagenesis experiments, does not interfere with 43_S0008 binding to group 1 and group 2 HAs (Figure S4A). Further, while the W21 residue of HA2 is targeted by CDR H2 I53 and F54 in canonical human IGHV1-69 bnAbs,^12^^,^^13^^,^^14^ the only interaction between the Fab 43_S0008 CDR H2 region and HA occurs between I53 and S298 of HA1 (Figure 3C; Table S3). Collectively, these data demonstrate that Fab 43_S0008 engages the central stem epitope similarly to other human IGHD3-9 antibodies that encode the LXYFXWL CDR H3 motif.

To better characterize whether the CDR H2 F54I/L substitution was stochastic or a necessity in gaining cross-group reactivity, we reverted the I/L54 residue back to phenylalanine for three IGHV1-69 + IGHD3-9 mAbs. Although we were unable to produce 43_S0008 with I54F due to the absence of protein expression, two other IGHV1-69 + IGHD3-9 mAbs, 43_S0035 and 43_S0047 with L54F and I54F, respectively, showed no change in binding to group 1 HAs; however, the substitutions severely impaired binding to group 2 HAs (Figure S4B), suggesting that the F54I/L substitution was critical in gaining cross-group reactivity for the IGHV1-69 + IGHD3-9 mAbs. This is supported by *in silico* analysis, which predicted that F54 in CDR H2 may cause steric clashes with L100 in CDR H3, potentially compromising antibody stability and/or requiring a reorganization of the CDR H3 loop to accommodate residue L100, which is part of the paratope (Figure S4C).

### Structural comparison of Fab 43_S0008 to canonical human IGHV1-69- and IGHD3-9-derived bnAbs

**Figure 4:**
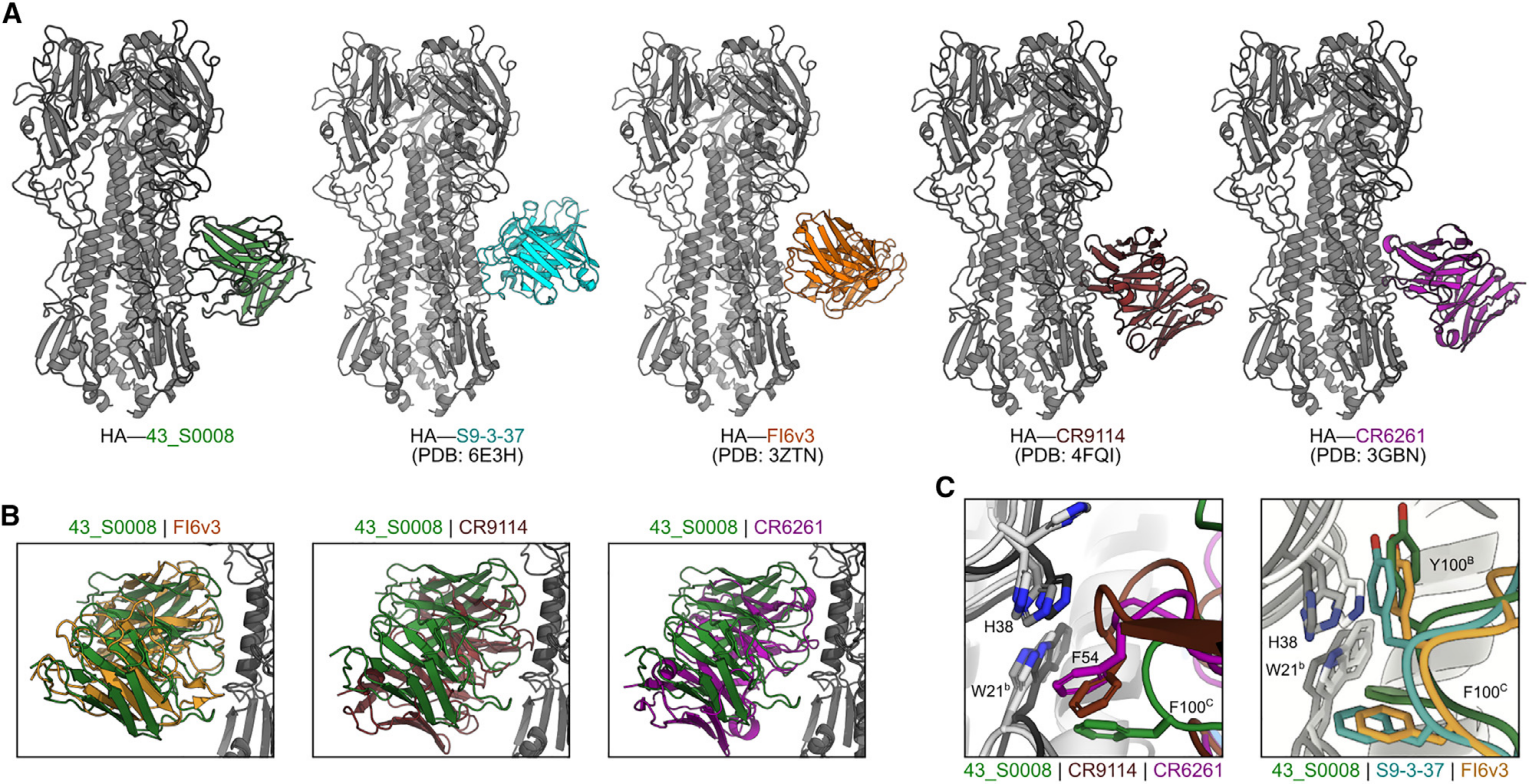
Structural comparison of 43_S0008 with human IGHV1-69- and IGHD3-9-derived bnAbs (A) HA recognition of IGHV1-69- and IGHD3-9-derived antibodies. HA trimer is colored gray and antibodies are displayed in different colors. The name of the antibody is colored according to the structure and PDB IDs as indicated. (B) Overlay of Fab 43_S0008 with human IGHD3-9-derived FI6v3 and IGHV1-69-derived bnAbs CR9114 and CR6261. (C) Close-up view of key interactions surrounding the W21^b^ residue in HA2. Comparison of 43_S0008 (green) with human IGHV1-69 bnAbs CR9114 and CR6261 (left) and IGHD3-9 bnAbs FI6v3 and S9-3-37 (right).

To better understand the mode of HA binding of the IGHV1-69 and IGHD3-9 mAbs, we compared the 43_S0008−HA structure to the structures of two known human IGHV1-69-derived bnAbs, group 1-specific CR6261^40^ and cross-group-reactive CR9114,^28^ as well as IGHD3-9-derived group 1-specific S9-3-37^33^ and cross-group-reactive FI6v3^32^ (Figure 4A). It is noteworthy that the heavy and light chains of S9-3-37 are positioned oppositely compared to the other antibodies. When we superposed Fab 43_S0008 to CR6261, CR9114, or FI6v3, we found that 43_S0008 resembled the overall structure of FI6v3, including the positioning of the CDR loops (Figure 4B). Specifically, the key CDR H3 residues Y100^B^ and F100^C^ of the three IGHD3-9-derived mAbs that mediate interaction with W21 are almost perfectly aligned, while this W21 interaction was mediated by CDR H2 residues (I53 and F54) for the IGHV1-69 mAbs^14^ (Figure 4C). In summary, Fab 43_S0008 resembles the IGHD3-9 bnAb FI6v3 in its angle of approach and superposition of the antibody CDR loops.

### 43_S0008 prophylaxis confers protection from H1N1 and H7N9 influenza virus challenges in mice

**Figure 5:**
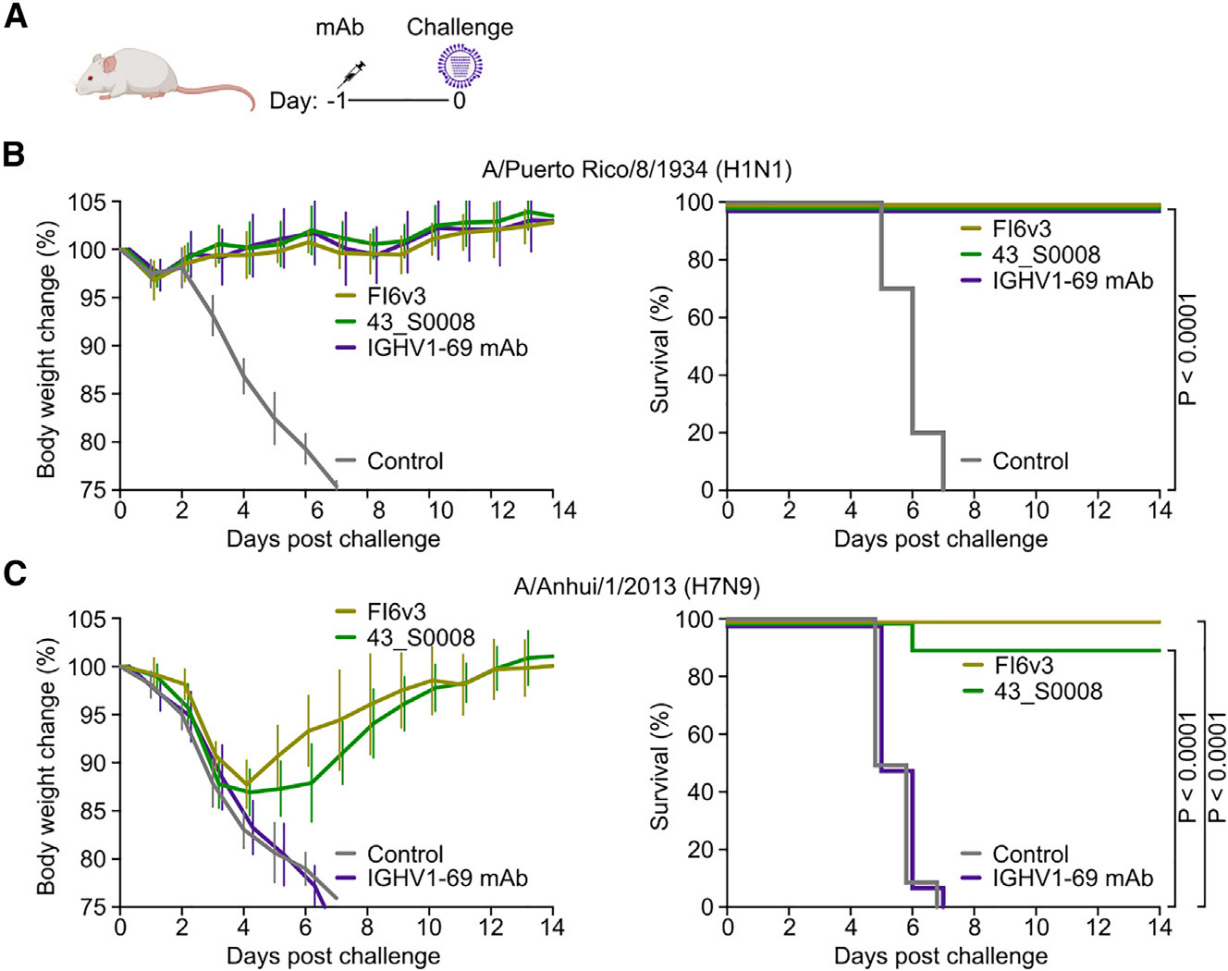
Antibody prophylaxis with IGHV1-69 + IGHD3-9 mAb 43_S0008 protects mice from lethal H1N1 and H7N9 influenza virus challenges (A) Experimental design of pre-exposure antibody prophylaxis in mice. BALB/c mice (*n* = 10) were given 43_S0008, FI6v3, or human IGHV1-69 bnAb at 10 mg kg^−1^ intraperitoneally 24 h prior to intranasal infection of A/Puerto Rico/8/1934 (H1N1) or A/Anhui/1/2013 (H7N9) virus. The human canonical IGHV1-69 mAbs CR6261 and 315-02-1H01 were used as comparators in the H1N1 and H7N9 challenges, respectively. Control mice received PBS instead of mAbs. (B and C) Changes in body weight (%) and survival curves are shown for H1N1 (B) and H7N9 (C) challenges. Body weight changes are plotted as mean ± SD of each treatment group. Multi-group comparison of Kaplan-Meier survival curves was performed with Mantel-Cox log rank test with Bonferroni correction.

To assess the protective efficacy of the IGHV1-69 + IGHD3-9 mAbs induced by the H7ssF-H1ssF-H1ssF immunization regimen, we chose 43_S0008 as our representative antibody in a passive transfer study. Mice were administered mAbs (10 mg kg^−1^) and challenged 24 h later with a lethal dose of A/Puerto Rico/8/1934 (H1N1) or AN13 (H7N9) virus (Figure 5A). For H1N1 challenge, all control mice receiving PBS succumbed to infection and were euthanized between days 5 and 7 post-challenge, whereas none of the mice receiving 43_S0008, human canonical IGHV1-69 mAb CR6261, or control cross-group-reactive bnAb FI6v3 suffered any weight loss, and all survived (Figure 5B). In contrast, all mice in the PBS and human canonical IGHV1-69 mAb 315-02-1H01^10^ groups succumbed to infection and were euthanized by day 7 after a lethal challenge with H7N9 virus infection (Figure 5C). Other groups of mice receiving either 43_S0008 or FI6v3 exhibited weight loss (∼12% of initial weight) in the first 4 days, but all recovered afterward and survived except for one animal in the 43_S0008 group (Figure 5C). Together, these results show that 43_S0008 conferred near-complete protection against both H1N1 (100%) and H7N9 (90%) virus challenges, as did FI6v3, which is one of the best-in-class cross-group-reactive bnAbs. In contrast, the canonical group 1-specific IGHV1-69 mAb was unable to confer protection against the group 2 H7N9 challenge, as would be expected. These results highlight that 43_S0008 and potentially other IGHV1-69 + IGHD3-9 mAbs distinctively confer protection against influenza viruses harboring both group 1 and group 2 subtype HAs, unlike the stereotypic IGHV1-69 mAbs.

### IGHV1-69 + IGHD3-9 and IGHD3-9-derived sequences in the human BCR repertoire

**Figure 6:**
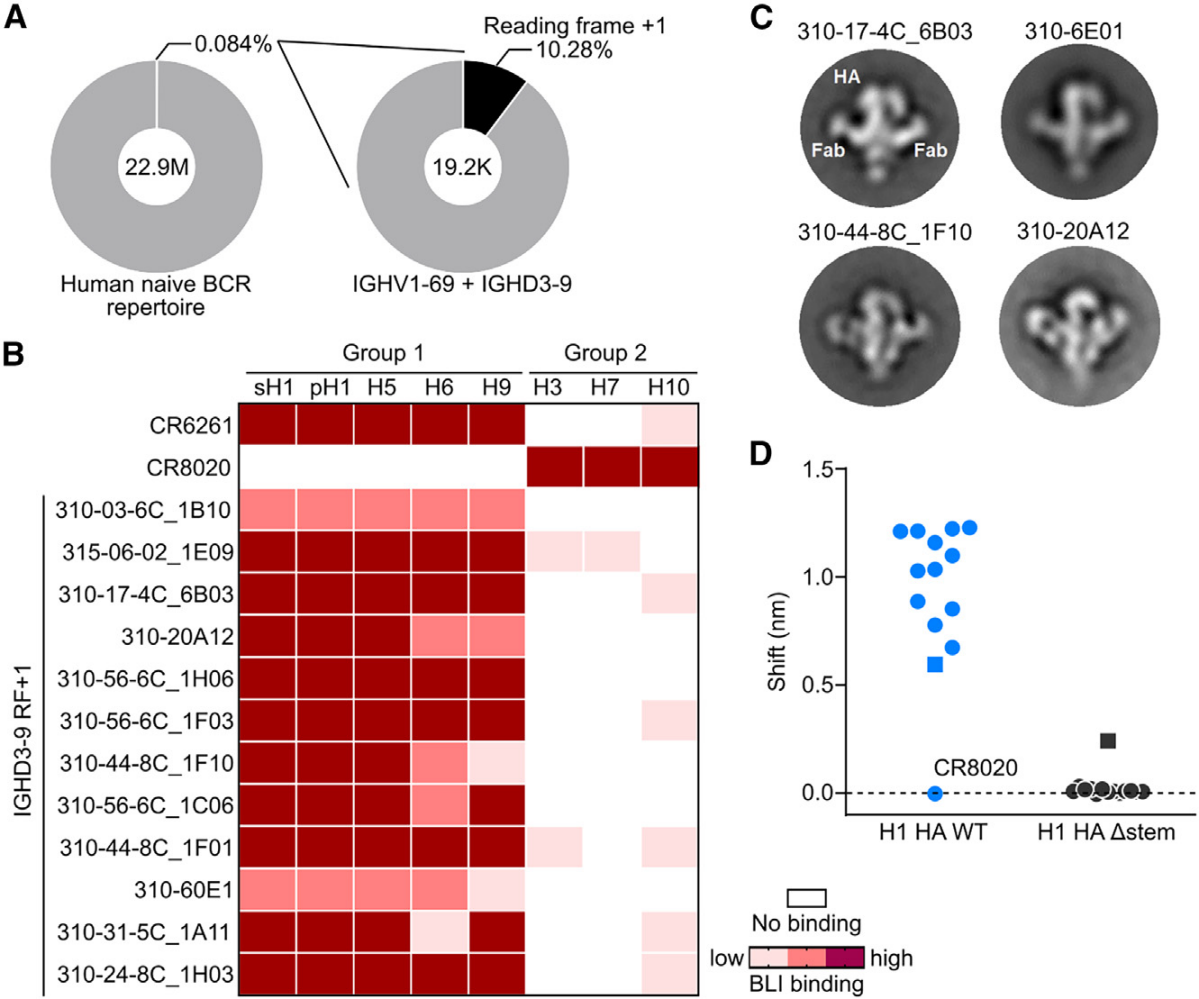
Characterization of IGHD3-9-derived antibodies in humans (A) Frequency of the IGHV1-69 + IGHD3-9 BCR sequences in a total of 22,860,000 sequences collected from three healthy subjects reported in DeWitt et al.^41^ (B) Characterization of human IGHD3-9-derived mAbs isolated from the VRC 310 and VRC 315 studies (ClinicalTrials.gov: NCT01086657 and NCT02206464). HA-binding profiles of the IGHD3-9-derived mAbs against various group 1 and group 2 HAs by BLI. White, pink, salmon, and cayenne indicate no binding, low binding, medium binding, and strong binding, respectively. (C) Negative-stain EM two-dimensional (2D) class averages of four representative human IGHD3-9-derived mAbs in complex with H1 HA. (D) Epitope specificity of human IGHD3-9-derived mAbs. HA binding of the IGHD3-9-derived mAbs to WT H1 HA and H1 Δstem HA assessed by BLI. One antibody (310-03-6C_1B10), which was insensitive to Δstem HA, is denoted with square symbols.

We next assessed the relative frequency of IGHV1-69 + IGHD3-9 sequences in the human naive B cell repertoire. We analyzed a total of ∼22.8 million sequences of naive B cell repertoire sorted based on the phenotype of CD3^−^ CD19^+^ CD27^−^ IgD^+^ IgM^+^ from three healthy adult subjects in the public database.^41^ The frequency of the IGHV1-69 + IGHD3-9 sequences in the naive repertoire is 0.084%, 10% of which have their IGHD3-9 sequence in RF+1 (Figure 6A). IGHV1-69 + IGHD3-9 sequences were also found in various memory B cell repertoires through the Observed Antibody Space Database,^42^ although very infrequently.^43^^,^^44^ While the combination of IGHV1-69 + IGHD3-9 RF+1 is apparently rare in human B cell repertoires, the contributions of IGHV1-69 to mediate HA interactions are negligible in our 43_S0008-HA structure. Therefore, we next expanded the scope to investigate a vaccine-elicited HA-specific B cell repertoire to find sequences that carry the IGHD3-9 RF+1 motif. We found several BCR sequences that match the criteria derived from the two human phase 1 studies evaluating experimental H5N1 and H7N9 vaccines conducted at the Vaccine Research Center, NIAID, NIH (VRC 310^45^ and VRC 315^46^ registered as ClinicalTrials.gov: NCT01086657 and NCT02206464, respectively). Twelve representative sequences were selected from nine individuals that were derived from nine different IGHV genes with IGHD3-9 RF+1 paired with various kappa and lambda light chains. We found that all tested IGHD3-9 RF+1 mAbs bound group 1 HAs, including H1, H5, H6, and H9, and a few of them had detectable binding to group 2 HAs (Figure 6B). This preferential group 1 HA binding is consistent with FI6v3, as it loses group 2 HA binding when somatically mutated residues are reverted.^32^ The human IGHD3-9 RF+1 mAbs target the central HA stem epitope and are sensitive to an added glycan at position 45 in the HA2 (Δstem HA), analogous to other central stem-targeting mAbs (Figures 6C and 6D). Together, these results suggest that cross-reactive IGHD3-9 RF+1 antibody responses can be actively induced in humans by immunization and that vaccine-inducible IGHD3-9 RF+1 mAbs share some key characteristics with the ones characterized in this study.

## Discussion

Germline-encoded motifs are often crucial for generating bnAbs to the sites of vulnerability and designing effective vaccines and/or vaccine regimens to elicit such bnAb responses. While it is critical to understand how to induce this hardwired immunity effectively and predictively at the conserved sites of vulnerability on hypervariable pathogens such as the influenza virus, HIV-1, and SARS-CoV-2, studying germline-targeting vaccines has been challenging due to the lack of adequate animal models to recapitulate human immunogenetics. In this study, we used a transgenic IGHV1-69 mouse model that exhibits fully humanized CDR H3 diversity in the IGHV1-69 backbone to address two key points related to the elicitation of bnAbs directed to the central stem epitope on influenza virus HA. Firstly, we evaluated the ability of a group 2 HA stem immunogen to reshape antibody responses in the face of a heavily group 1-biased IGHV1-69 repertoire. Although the intrinsic mode of group 1 HA recognition by IGHV1-69 involves a hydrophobic CDR H2 tip, this mode of recognition is incompatible with group 2 HAs due to the differences in glycosylation patterns between group 1 and group 2 HAs. We found that group 2 HA stem priming made the immune system pivot from a CDR H2-centric recognition to a CDR H3-centric recognition, which results in expanding the breadth of specificity beyond group 1 HAs through employing another germline-encoded motif—RYFDW encoded by IGHD3-9 RF+1—in the IGHV1-69 environment. This not only highlights the adaptability of the stochastic V-D-J repertoire but also informs potential vaccine regimens to selectively engage a more broadly cross-reactive B cell repertoire in a highly competitive hierarchical environment. Secondly, we defined the structural basis for HA recognition by the IGHD3-9 RF+1 motif that is vividly reminiscent of the interactions made by the human bnAb FI6v3. However, unlike FI6v3, the IGHD3-9 RF+1 antibodies we isolated from IGHV1-69 mice in this study did not cross-react with H3 HA. Although FI6v3 and its original form, FI6, have exceptional cross-group reactivity, its germline-reverted form (FI6-GL) loses the ability to bind group 2 HAs, including H3.^32^ Further, FI6/370-BP, which represents an intermediately matured clonal member of FI6, shows cross-reactivity with H7 but has negligible binding to H3, suggesting that cross-reactivity to group 2 HA is acquired stepwise through somatic hypermutation.^32^ Similar partial cross-group reactivity was seen in the macaque bnAb 789-203-3C12, which was elicited in an influenza-naive macaque through immunization with HA stem immunogens.^47^ Our results, along with those of these other studies, suggest that the induction of broadly cross-group antibodies may require preexisting influenza immunity and/or repeated exposure to influenza virus or vaccine. While human bnAbs that use the IGHD3-9 RF+1 motif to target the central stem epitope, including FI6v3, have been described previously,^10^^,^^32^^,^^33^ our results broaden this knowledge by showing that the IGHD3-9 RF+1 bnAb response is readily vaccine inducible in a total of nine different individuals with nine different IGHV genes paired with IGHD3-9 RF+1. Our findings further underscore the broad applicability and generalizability of IGHD3-9 RF+1 bnAbs.

Human Ig transgenic mouse models have been used to study germline-targeting vaccine candidates for HIV-1 and other pathogens for eliciting certain antibody responses and their developmental pathways.^48^^,^^49^^,^^50^^,^^51^ Notably, a promising HIV-1 vaccine candidate, eOD-GT8-60mer, extensively characterized in the transgenic mouse model, has undergone evaluation in human clinical trials,^52^^,^^53^ registered with ClinicalTrials.gov: NCT03547245, NCT05001373, and NCT05414786, validating the usefulness of transgenic mouse models in evaluating vaccine candidates. In addition, transgenic mice also offer the opportunity to study subdominant immune responses that would otherwise be overshadowed under physiological conditions. In the context of influenza HA-stem-specific immunity, human IGHV1-69 mice have been used to reproduce canonical hardwired immunity in humans enabled by the IGHV1-69 CDR H2 motif,^29^ as well as to study basic immunological questions about the impact of allelic variations of IGHV1-69 on immune response and immunopathology.^30^ We used this IGHV1-69 mouse model to study the effect of an immunization regimen on the immune repertoire and the non-canonical bnAb development pathway through the IGHD3-9 RF+1 motif.

Influenza HA-stem-directed public antibodies have revealed biased usages of certain germline IGHV genes such as IGHV1-69, IGHV6-1, and IGHV1-18. Yet, some of these known public bnAb classes require CDR H3 motifs that are encoded by specific IGHD genes, such as the IGHD3-3-encoded IFGVV motif of the IGHV6-1 class and the IGHD3-9 RF+2-encoded ILTG motif of one of the two IGHV1-18 classes.^10^^,^^33^^,^^54^ Intriguingly, HIV-1 envelope (Env)-specific BG18-like bnAbs that use the IGHD3-3-encoded IFGVV motif have been shown to make critical contacts with their epitope in a highly CDR H3-dependent fashion, and this class of antibodies are recurrently found in multiple individuals with different IGHV genes.^54^ The recurrent IGHD3-9-centric mode of HA stem recognition in humans has also been previously reported by Wu et al.^33^ The authors proposed that the IGHD3-9 gene provides germline-encoded immunity against influenza viruses by employing either RF+1 (RYFDW)- or RF+2 (ILTG)-encoded motifs.

In conclusion, our findings show that under certain immunization conditions, the default mode of binding of IGHV1-69 B cells can be subverted, and the same HA central stem supersite can be targeted through another hardwired motif encoded by IGHD3-9 RF+1. These findings offer a framework for vaccine regimens that engage and improve the B cell repertoire, promoting cross-group reactivity and providing protection against a broader range of influenza viruses.

### Limitations of the study

This study demonstrates that IGHV1-69-derived antibodies can gain cross-group reactivity by pairing with the IGHD3-9 gene, which serves as a primary determinant for HA recognition. Our IGHV1-69 mouse model allows us to study the IGHV1-69 repertoire in the absence of competition from all the other clones; however, B cells harboring both IGHV1-69 and IGHD3-9 genes might be outcompeted by other B cells in humans upon infection, vaccination, or clonal deletion. Another limitation of this mouse model is that it utilizes murine light chains instead of human light chains. Additionally, the use of the A/Puerto Rico/8/1934 influenza virus for challenge studies rather than a more contemporary post-2009 H1N1 virus, such as A/California/04/2009, limits the applicability of the findings to currently circulating influenza strains. Preexisting immunity upon repeated exposure to influenza viruses has a major impact on shaping the immune repertoire in humans. A more complex model or, eventually, a human study is needed to fully understand the IGHD3-9 RF+1-centric bnAb development pathway and test candidate vaccines that would elicit such bnAb responses in humans.

## Resource availability

### Lead contact

Further information and requests for resources and reagents should be directed to and will be fulfilled by the lead contact, Masaru Kanekiyo (kanekiyom@nih.gov).

### Materials availability

All materials relevant to this manuscript are available from the lead contact with a completed materials transfer agreement.

### Data and code availability

The PDB IDs and Electron Microscopy Data Bank (EMDB) IDs for the H1 HA in complex with the mAb 43_S0008 have been deposited into the RCSB PDB (https://www.rcsb.org) under PDB: 9B2M and to the EMDB database (https://www.ebi.ac.uk/emdb/) under EMDB: EMD-44112.

This study does not report original code.

Any additional information required to reanalyze the data reported in this paper is available from the lead contact upon request.

## Acknowledgments

The authors thank R. Koup and members of the VRC Influenza Program for helpful discussions and H. Andersen Elyard, M. Porto, and other Parklawn Drive Facility staff (Bioqual, Inc.) for challenge studies. We thank B. Anderson and H.L. Turner for cryo-EM data collection support and C. Bowman and J.C. Ducom for computational support. This work was supported, in part, by the Intramural Research Program of the Vaccine Research Center, 10.13039/100000060NIAID, NIH (M.K.) and federal funds from the 10.13039/100012728Frederick National Laboratory for Cancer Research, NIH, under contract no. HHSN261200800001 (Y.T. and T.S.). D.L. was supported by R01 funding from the 10.13039/100000002NIH (AI137057, AI153098, and AI155447). A.T.d.l.P. is a recipient of NWO Rubicon grant 45219118, and R.d.P.F.R. is supported by the São Paulo Research Foundation (FAPESP grant number 2019/20772-4). Cryo-EM was supported by 10.13039/100000865Bill and Melinda Gates Foundation grants OPP1170236 and INV-004923.

## Author contributions

Conceptualization, S.A. and M.K.; formal analysis, S.A., M.S., R.d.P.F.R., A.T.d.l.P., S.B.-B., S.F.A., A.B.W., D.L., and M.K.; investigation, S.A., M.S., R.d.P.F.R., A.T.d.l.P., L.R., S.B.-B., R.A.G., Y.T., T.S., S.M.M., J.L., S.F.A., R.M.B., D.R., N.L., A.B.W., D.L., and M.K.; resources, A.C., R.M.B., D.R., and N.L.; writing—original draft, S.A. and M.K.; writing—review & editing, all authors; supervision, B.S.G., A.B.W., D.L., and M.K.; funding acquisition, B.S.G., A.B.W., D.L., and M.K.

## Declaration of interests

S.M.M., B.S.G., and M.K. are listed as inventors of patents and patent applications on vaccine immunogens used in this study filed by the NIH, US Department of Health and Human Services.

## STAR★Methods

### Key resources table

**Table:** 

REAGENT or RESOURCE	SOURCE	IDENTIFIER
**Antibodies**		

43_S0001	This paper	N/A
43_S0002	This paper	N/A
43_S0005	This paper	N/A
43_S0008	This paper	N/A
43_S0009	This paper	N/A
43_S0010	This paper	N/A
43_S0011	This paper	N/A
43_S0012	This paper	N/A
43_S0013	This paper	N/A
43_S0015	This paper	N/A
43_S0016	This paper	N/A
43_S0017	This paper	N/A
43_S0019	This paper	N/A
43_S0020	This paper	N/A
43_S0022	This paper	N/A
43_S0024	This paper	N/A
43_S0029	This paper	N/A
43_S0031	This paper	N/A
43_S0032	This paper	N/A
43_S0034	This paper	N/A
43_S0035	This paper	N/A
43_S0036	This paper	N/A
43_S0037	This paper	N/A
43_S0038	This paper	N/A
43_S0039	This paper	N/A
43_S0040	This paper	N/A
43_S0044	This paper	N/A
43_S0045	This paper	N/A
43_S0046	This paper	N/A
43_S0047	This paper	N/A
43_S0048	This paper	N/A
CR6261	Produced in house, Throsby et al.^40^	N/A
CH65	Produced in house, Whittle et al.^55^	N/A
CR8020	Produced in house, Ekiert et al.^56^	N/A
315-02-1H01	Produced in house, Andrews et al.^10^	N/A
310-03-6C_1B10	Produced in house, Andrews et al.^10^	N/A
315-06-02_1E09	Produced in house, Andrews et al.^10^	N/A
310-17-4C_6B03	Produced in house, Andrews et al.^10^	N/A
310-20A12	Produced in house, Andrews et al.^10^	N/A
310-56-6C_1H06	Produced in house, Andrews et al.^10^	N/A
310-56-6C_1F03	Produced in house, Andrews et al.^10^	N/A
310-44-8C_1F10	Produced in house, Andrews et al.^10^	N/A
310-56-6C_1C06	Produced in house, Andrews et al.^10^	N/A
310-44-8C_1F01	Produced in house, Andrews et al.^10^	N/A
310-60E1	Produced in house, Andrews et al.^10^	N/A
3100-31-5C_1A11	Produced in house, Andrews et al.^10^	N/A
310-24-8C_1H03	Produced in house, Andrews et al.^10^	N/A
Anti-CD3 PE-Cy7	Biolegend	Cat#100320; RRID:AB_312685
Anti-CD4 Alexa Fluor 700	Biolegend	Cat#557975
Anti-CD19 BV421	Biolegend	Cat#115549; RRID:AB_2563066
Anti-IgM BV605	Biolegend	Cat#406523; RRID:AB_2563358
Anti-IgG PerCP-Cy5.5	Biolegend	Cat#405314; RRID:AB_10662053
Sheep anti-human IgG-HRP	GE Healthcare	Cat#NA933
Sheep anti-mouse IgG-HRP	GE Healthcare	Cat#NA931

**Bacterial and virus strains**		

H1N1 reporter virus	Produced in house, Creanga et al.^37^	N/A
H5N1 reporter virus	Produced in house, Creanga et al.^37^	N/A
H7N9 pseudovirus	Produced in house, Kong et al.^38^ and Yang et al.^39^	N/A
H10N8 pseudovirus	Produced in house, Kong et al.^38^ and Yang et al.^39^	N/A

**Biological samples**		

Human PBMCs	NCT01086657; NCT02206464	N/A

**Chemicals, peptides, and recombinant proteins**		

Sigma Adjuvant System	Sigma-Aldrich	Cat#S6322
H1ssF	Produced in house, Yassine et al.^6^	N/A
H7ssF	Produced in house, Corbett et al.^7^	N/A
RLT lysis buffer	Qiagen	Cat#79216
LIVE/DEAD Fixable Aqua Dead Cell Stain	Thermo Fisher	Cat#L34957
H1 A/New Caledonia/20/1999 HA	Produced in house, Wei et al.^57^	N/A
H1 A/New Caledonia/20/1999 Δstem HA	Produced in house, Wei et al.^57^	N/A
H7 A/Anhui/1/2013 HA	Produced in house, Boyoglu-Barnum et al.^58^	N/A
LysC	New England Biolabs	Cat#P8109S
Protease inhibitor cocktail	MilliporeSigma	Cat#P8849
Luciferase Assay System	Promega	Cat#E1500
MiSeq Reagent Kit, V2 500 cycles	Illumina	Cat#MS-102-2003

**Deposited data**		

Coordinates of HA in complex with 43_S0008	this study	PDB: 9B2M
Cryo-EM map of HA in complex with 43_S0008	this study	EMDB: EMD-44112

**Experimental models: Cell lines**		

Expi293F cells	Thermo Fisher	Cat#A14527
MDCK-SIAT1-PB1	Creanga et al.^37^	N/A
HEK293T-PB1	Creanga et al.^37^	N/A

**Experimental models: Organisms/strains**		

IGHV1-69 transgenic mouse	Bristol Myers Squibb, Sangesland et al.^29^	N/A
BALB/cAnNHsd female mice	Envigo	Cat#047; RRID:IMSR_ENV:HSD-047

**Oligonucleotides**		

Partially degenerate human/mouse V region specific primers	Sangesland et al.^29^	N/A
Reverse primers against the heavy or light chain constant regions	Sangesland et al.^29^	N/A

**Software and algorithms**		

Biorender	biorender.com	N/A
Prism v10	GraphPad https://www.graphpad.com	N/A
FlowJo v10.6.2	FlowJo LLC https://www.flowjo.com	N/A
WebLogo	https://weblogo.berkeley.edu; Crooks et al.^59^	N/A
Smart-Seq2	Trombetta et al.^60^	N/A
PandaSeq	Masella et al.^61^	N/A
Leginon	Suloway et al.^62^	N/A
MotionCor2	Zheng et al.^63^	N/A
cryoSPARC v3.1.0	Punjani et al.^64^	N/A
ABodyBuilder	Leem et al.^65^	N/A
Rosetta	Wang et al.^66^	N/A
Coot	Emsley and Crispin^67^	N/A
EMRinger	Barad et al.^68^	N/A
MolProbity	Williams et al.^69^	N/A
UCSF ChimeraX	Pettersen et al.^70^	N/A

**Other**		

Octet HIS1K biosensors	Sartorius	Cat#18-5120
Octet HIS2 biosensors	Sartorius	Cat#18-5114
Galanthus nivalis lectin, Agarose bound	Vector Labs	Cat# AL-1243-5
Ni Sepharose Excel	Cytiva	Cat#GE17371201
Superose 6 increase 10/300 GL	Cytiva	Cat#GE29-0915-96
Superdex 200 increase 10/300 GL	Cytiva	Cat#GE28-9909-44
1.2/1.3 300 mesh holey carbon grids	Quantifoil	Cat#Q225CR-06

### Experimental model and study participant details

The transgenic mice used in this study have been previously characterized.^29^^,^^30^^,^^31^ Briefly, mice were generated such that the antibody heavy chain locus was constrained to a single human IGHV gene with diverse human IGHD and IGHJ segments, allowing for human-like recombination and CDR H3 diversification. In this study, all B cell receptors were constrained to the human IGHV1-69^∗^01 with the light chain repertoire provided by the native C57BL/6 murine kappa and lambda locus. Animals were maintained within the Ragone Institute’s HPPF barrier facility and experiments were conducted with IACUC approval (MGH protocol 2014N000252). Both male and female mice, aged 6–10 weeks, were used. Use of these animals requires an MTA with Bristol Myers Squibb. Human PBMC samples used to isolate mAbs were obtained from VRC 310 and VRC 315, single-site, phase 1, open-label, randomized clinical trials conducted at the National Institutes of Health (NIH) Clinical Center by the NIAID VRC (Clinicaltrial.gov NCT01086657 and NCT02206464).^45^^,^^46^ These studies were approved by the NIAID Intramural Institutional Review Board. U.S. Department of Health and Human Services guidelines for conducting clinical research were followed.

### Method details

#### Expression and purification of immunogens and antibodies

The plasmids of IGHD3-9 sequences obtained from the next-generation sequencing of B cells isolated two weeks after the third immunization, as well as the F54A, R100^A^A, Y100^B^A, F100^C^A, D100^D^A, W100^E^A mutants of mAbs 43_S0008, 43_S0035, 43_S0047 were synthesized by GenScript. The IGHD3-9 mAbs, the mutant mAbs, and control mAbs were expressed in Expi293 cells (Life Technologies). HA ectodomains from A/New Caledonia/20/1999 (NC99) or A/Anhui/1/2013 were expressed in 293F cells (Life Technologies) and purified as soluble wild-type (WT) and Δstem HA (I45R/T49R or 45 glycan) trimers, or as ferritin nanoparticles displaying 8 copies of stabilized HA stem trimer (ssF), as performed previously.^6^^,^^34^^,^^71^ All proteins were purified by affinity chromatography (*Galanthus nivalis* lectin for the nanoparticles, nickel beads for the trimers and protein A for mAbs), using previously described methods.^6^ Assembly of trimers and nanoparticles was assessed by gel filtration with a Superdex 200 10/300 column and Superose 6 increase 10/300 GL column (GE Healthcare), respectively. To make Fabs, IgG was digested with endoproteinase Lys-C (New England Biolabs) overnight at room temperature (RT). The reactions were assessed by SDS-PAGE and, upon completion, were quenched by the addition of protease inhibitor cocktail (MilliporeSigma), and Fc and Fab antibodies were separated by protein A affinity chromatography. For B cell probes, full length versions of soluble trimeric HA ectodomains from H1 (NC99) and H5 (A/Indonesia/05/2005) were generated and purified as described previously.^25^^,^^29^ HA probes were biotinylated and labeled with streptavidin-phycoerythrin (H1-PE) or streptavidin-allophycocyanin (H5-APC), producing fluorescent tetramers of HA trimers. The Y98 mutation was included to prevent sialic acid binding.^25^ All recombinant HA proteins were quality controlled by size exclusion chromatography and by binding to the conformational antibodies CR6261 and CH65.^40^^,^^55^

#### Immunization regimens

IGHV1-69 transgenic mice were primed (day 0) with 15 μg of H7ssF followed by sequential vaccination with H1ssF at days 21 and 42. All animals were pre-bled prior to each experiment and bled 14 days following the last exposure. Immunizations were administered via the intraperitoneal route with 100 μL of inoculum containing 50% w/v Sigma Adjuvant System.

#### Flow cytometry

Single cell suspensions of mouse splenocytes were obtained using a 70 μM strainer treated with ACK lysis buffer to remove red blood cells. Cells were stained with the following antibodies: CD3 PE/Cy7 (Biolegend, cat# 100320), CD4 Alexa Fluor 700 (Biolegend, cat# 557975), CD19 BV421 (Biolegend, cat# 115549), IgM BV605 (Biolegend, cat# 406523), IgG PerCP/Cy5.5 (Biolegend, cat# 405314) with the addition of 0.25 μg each of H1-PE and H5-APC probes. Cells were also stained with Aqua Live/Dead and Calcein. Antigen specific B cells were defined as H1^+^H5^+^ within CD3^−^ CD19^+^ IgM^−^ IgG^+^ and isolated by FACS into plates containing RLT lysis buffer with 1% β-mercaptoethanol. Downstream analysis of the data was performed using the FlowJo software v10.6.2 (FlowJo).

#### BCR sequencing

Single cell BCR sequencing was performed as described previously.^29^ Briefly, whole transcriptome amplification (WTA) products were generated from H1^+^H5^+^ sorted B cells using the Smart-Seq2 protocol.^60^ Heavy and light chain BCR sequences were amplified using a pool of partially degenerate V region specific primers encompassing all human IGHV or mouse IGLV and IGKV segments and reverse primers against the heavy or light chain constant regions. These primers were attached to the Illumina P7 and P5 sequences. Step out PCR was performed to add cellular barcodes and Illumina sequencing adaptors (based on Nextera XT Index Adapters). Samples were pooled and sequenced using paired-end V2-500 cycle Illumina Miseq System. After demultiplexing, sequences were paired and overlapping sequence reads were reconstructed using PandaSeq^61^ and then aligned against the human IMGT database^72^ with error correction using MigMAP. Consensus sequences for each single cell was determined by collapsing all reads with the same CDR3 sequence and calling the top heavy and light chain sequences by frequency.

#### ELISA

ELISA was done to assess anti-HA antibodies in response to HAssF immunizations. The plates were coated with 2 μg well^−1^ HAssF, HAss (stabilized stem-only HA) and full-length HA antigens overnight at 4°C. The plates were then incubated with mAbs with dilution series starting at 100 nM, which were serially diluted 1:4. For testing of the mouse sera, the H1 and H7 HA antigens represented A/New Caledonia/20/1999and A/Anhui/1/2013, respectively. Mouse serum serial dilutions started at 1/20 and were serially diluted at 1:5. Plates were washed with PBST and incubated with either goat anti-human IgG-HRP or sheep anti-mouse IgG-HRP (GE Healthcare) at 1/5000 dilution in PBS. Plates were developed using TMB and quenched with 1N sulfuric acid. The absorbance values (optical density read at 450 nm) were plotted using the GraphPad Prism (v10.6.2).

#### Biolayer interferometry

All biosensors were hydrated in PBS before use. Recombinant HA proteins were immobilized for 10 min either on HIS1K or HIS2 biosensors (Sartorius) through their hexahistidine tag. After briefly dipping in assay buffer (1% BSA in PBS), the biosensors were dipped in IgG for 5 min. Biosensors were then dipped in assay buffer to allow IgG/Fab to dissociate from HA for 5 min. All assay steps were performed at 30°C with agitation set at 1,000 r.p.m. in the Octet HTX instrument (fortéBio). Correction to subtract non-specific baseline drift was carried out by subtracting the measurements recorded for a blank sensor. Data analysis were carried out using Octet analysis software (version 12.0). The heatmaps were drawn in the GraphPad Prism (v10.6.2).

#### Microneutralization assays

All reporter viruses were prepared as described previously.^37^ Briefly, H1N1 virus was made with a modified PB1 segment expressing the TdKatushka reporter gene (R3ΔPB1) and propagated in MDCK-SIAT-PB1 cells, while H5N1 reporter virus was made with a modified HA segment expressing the reporter (R3ΔHA) and produced in cells stably expressing H5 HA. Rescued viruses were propagated in MDCK-SIAT1-PB1 in the presence of TPCK-treated trypsin (1 μg mL^−1^, Sigma) at 34°C. Virus stocks were stored at −80°C. mAbs were serially diluted and incubated for 1 h at 37°C with pre-titrated viruses. Serum-virus mixtures were then transferred to 96-well plates (PerkinElmer), and 1.0 × 10^5^ MDCK-SIAT1-PB1 cells^37^^,^^73^ were added into each well. After overnight incubation at 37°C, the number of fluorescent cells in each well was counted automatically using a Celigo image cytometer (Nexcelom Biosciences). Neutralization curves were plotted with GraphPad Prism (v10.6.2) using a four-parameter nonlinear regression model.

#### Lentiviral pseudotype neutralization assays

Lentiviral pseudotype neutralization assays were carried out using luciferase-encoding lentiviruses pseudotyped with influenza HA and NA, as described previously.^38^^,^^39^ The HA and NA sequences used to generate the pseudoviruses were derived from A/Anhui/1/2013 (H7N9) and A/Jiangxi-Donghu/346-2/2013 (H10N8). Briefly, mAbs were serially diluted and incubated with pre-titrated HA pseudotyped viruses for 30 min at room temperature. Serum-pseudovirus mixtures were then transferred to 96-well white/black isoplates (PerkinElmer), and 293A cells (12,000 cells) were added into each well of the plate. After overnight incubation at 37°C, wells were supplemented with 100 μL of fresh Dulbecco’s Modified Eagle Medium including 5% fetal bovine serum (Fisher Scientific) and 5,000 units ml^−1^ penicillin-streptomycin (Gibco), and the plates were incubated in a static 37°C, 5% CO_2_, humidified incubator for 48 h. Cells were lysed with cell culture lysis buffer (Promega) and luciferase activity in the lysate was measured using Luciferase kit (Promega). Luminescence was measured with a Spectramax L luminometer (Molecular Devices). Neutralization curves were plotted with GraphPad Prism (v10.6.2) using a four-parameter nonlinear regression model.

#### Passive transfer

BALB/cAnNHsd female mice between 4 and 6 weeks of age were purchased from Envigo and were housed either in a conventional or specific pathogen free (SPF) animal facility on a 12-h light/dark cycle under ambient conditions with free access to food and water. Animals were randomly assigned to experimental groups. All experiments were done in accordance with approved guidelines, regulations, and protocols as determined and approved by the Institutional Animal Care and Use Committee at Bioqual, Inc., Rockville, MD. The research facility is AAALAC International accredited and standards for all animal care (acquisition, breeding, and experimental protocols), biosafety, and personnel occupational health and safety conform to all Federal, State and local regulations. Mice were given intraperitoneally 0.2 mg of purified monoclonal antibodies (approximately 10 mg kg^−1^). Twenty-four hours later, the mice were infected intranasally with 10 × LD_50_ of H1N1 (A/Puerto Rico/8/1934) or H7N9 (A/Anhui/1/2013) viruses at Bioqual. The mice were monitored twice daily for development of clinical signs of infection and weighed daily for 14 days. Any mice that lost 20% or more of their initial body weight were humanely euthanized.

#### Cryo-EM analysis

Fab 43_S0008 was complexed with 2 mg ml^−1^ of HA at a 1:3 M ratio (HA:Fab) for 30 min at room temperature (RT). The HA-Fab complex at a final concentration of 1 mg ml^−1^ was mixed with 0.5% Lauryl maltose neopentyl glycol (LMNG). QUANTIFOIL 1.2/1.3 300 mesh holey carbon grids were plasma cleaned for 7 s using the solarus advanced plasma cleaning system (Gatan) before loading the sample. Further, 3 μL of the sample were loaded onto the grid and plunge-frozen into nitrogen-cooled liquid ethane using the Vitrobot mark IV (Thermo Fischer Scientific). The settings for the Vitrobot were as follows: Temperature: 4°C, humidity: 100%, blotting time: 5.5 s, blotting force: 1 and wait time: 3.5 s.

Grids were loaded into a Titan Krios Cryo-Transmission Electron Microscope (Thermo Fisher Scientific) operating at 300 kV and equipped with K2 Summit direct electron detector camera (Gatan). The data was collected at a total cumulative dose of 50 e^−^ per Å^2^. Magnification was set at 29,000x with a resulting pixel size of 1.045 Å per pix at the specimen plane. Automated data collection was performed using Leginon software.^62^ The data collection details are presented in Table S1. The micrographs movie frames were aligned and dose-weighted using MotionCor2.^63^ In cryoSPARC v3.1.0^64^ patch CTF was applied and micrographs were manually curated. Further, 1,417,466 particles were picked from micrographs using template picker. The particles were then extracted and a few rounds of 2D classification were performed. Bad particle picks were eliminated and classes with Fab–HA complexes were subjected to a round of Non-Uniform Refinement. A low pass-filtered map of NC99 HA trimer was used as initial model for all 3D steps, to avoid initial bias. Two rounds of heterogeneous refinement were run using the trimer–Fab complex. The highest resolution classes were selected, 3D refined and postprocessed (C3 symmetry). In Chimera, HA trimer A/California/07/09 (PDB: 4M4Y) crystal structure was aligned with HA NC99 and used as initial model. The model for Fab 43_S0008 was built using ABodyBuilder.^65^ The post-processed map was used to model the final atomic models. Multiple rounds of Rosetta relaxed refinement^66^ and manual Coot refinement^67^ were performed. To validate the analysis, EMRinger^68^ and MolProbity^69^ were used. The final refined model was deposited to the Protein DataBank (PDB). All structural figures were generated using UCSF ChimeraX.^70^

### Quantification and statistical analysis

All statistical analyses were performed using the GraphPad Prism (v10.6.2). Sample sizes of animals and specific tests to determine statistical significance used are indicated in the corresponding figure captions.

## Supplemental information

Document S1. Figures S1–S4 and Tables S1–S3

Document S2. Article plus supplemental information
