# Supplementary material for: Modulating the immunodominance hierarchy of immunoglobulin germline-encoded structural motifs targeting the influenza hemagglutinin stem
Source: Cell Rep. 2024 Nov 22;43(12):114990. doi: 10.1016/j.celrep.2024.114990 (PMC11672684; doi:10.1016/j.celrep.2024.114990)
Supplement: Document S1. Figures S1–S4 and Tables S1–S3 [file mmc1.pdf]

Cell Reports, Volume *43*

Supplemental information

Modulating the immunodominance hierarchy of immunoglobulin germline-encoded structural motifs targeting the influenza hemagglutinin stem

Sila Ataca, Maya Sangesland, Rebeca de Paiva Fróes Rocha, Alba Torrents de la Peña, Larance Ronsard, Seyhan Boyoglu-Barnum, Rebecca A. Gillespie, Yaroslav Tsybovsky, Tyler Stephens, Syed M. Moin, Julia Lederhofer, Adrian Creanga, Sarah F. Andrews, Ralston M. Barnes, Daniel Rohrer, Nils Lonberg, Barney S. Graham, Andrew B. Ward, Daniel Lingwood, and Masaru Kanekiyo

Table S1. Binding profiles of the IGHD3-9 antibodies from the H7ssF-H1ssF-H1ssF immunization group against H1 and H5 HA antigens tested by Biolayer Interferometry, Related to Figure 2

Antibody	H1 HA	H5 HA
43_S0001	+	+
43_S0002	+	+
43_S0005	+	+
43_S0008	+	+
43_S0009	+	+
43_S0010	+	+
43_S0011	+	+
43_S0012	+	+
43_S0013	+	+
43_S0015	+	+
43_S0016	+	+
43_S0017	+	+
43_S0019	+	+
43_S0020	+	+
43_S0022	+	+
43_S0024	+	+
43_S0029	+	+
43_S0031	+	+
43_S0032	+	+
43_S0034	+	+
43_S0035	+	+
43_S0036	+	+
43_S0037	+	+
43_S0038	+	+
43_S0039	+	+
43_S0040	+	+
43_S0044	+	+
43_S0045	+	+
43_S0046	+	+
43_S0047	+	+
43_S0048	+	+

Table S2. Cryo-EM data collection and refinement statistics, Related to Figure 3

H1 NC99 HA–Fab 43_S0008 complex	
Microscope	Titan Krios
Voltage (kV)	300
Detector	K2 Summit DED
Recording mode	Counting
Magnification	29,000 ×
Movie micrograph pixel size	1.045 Å/pix
Number of frames per movie micrographs	40
Frame exposure (ms)	250
Movie micrograph exposure time (s)	9.56
Total dose (e^-/Å^2)	50
Grid type	QUANTIFOIL®1.2/1.3
Focus range (μm)	-0.8 to -1.4
Number of movie micrographs	40
Number of picked particles	1,417,466
Particles after 2D classification	190,711
Particles in the final map	190711
Map symmetry	C3
Map sharpening B-factor	117.9
Map Resolution (Å)	3.09
EMDB ID	EMD-44112
Residues	
Amino acids	2,187
Carbohydrates	15
RMSD Bonds	0.022 (97)
RMSD Angles	1.842 (97)
Ramachandran	
Outliers (%)	0
Allowed (%)	2.13
Favored (%)	97.87
Rotamers outliers (%)	0.11
Clash score	0.96
Molprobity score	1.08
EMRinger score	2.51
PDB ID	9B2M

Table S3. Contact residues between 43_S0008 and H1 HA trimer, Related to Figure 3

Region	Antibody	HA
CDRH1	R30	S298
	T31	I57
CDRH2	I53	S298
FR3	D72	N296
	E73	N296
	S74	K287
	N76	K287
CDRH3	R97	N46^{b}, N50^{b}
	L99	Q42^{b}, N46^{b}
	L100	I45^{b}, T49^{b}, V52^{b}
	Y100^{B}	H38, T325, D19^{b}, W21^{b}
	F100^{C}	D19^{b}, G20^{b}, W21^{b}, S40^{b}, I45^{b}
	W100^{E}	T41^{b}, D37^{b}
	L100^{F}	Q42^{b}
	P100^{G}	Q42^{b}
CDRL1	S27^{E}	Q38^{b}
	D28	Q42^{b}
	Y32	Q42^{b}

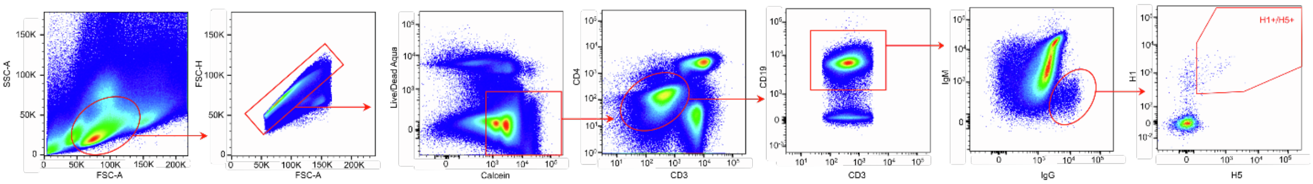

Figure S1. Flow cytometry gating scheme, Related to Figure 1

Gating strategy to isolate $H1^{+}H5^{+}$ B cells ($CD19^{+}$ $CD3^{-}$ $CD4^{-}$ IgM^{-} IgG^{+}) from spleen of immunized mice.

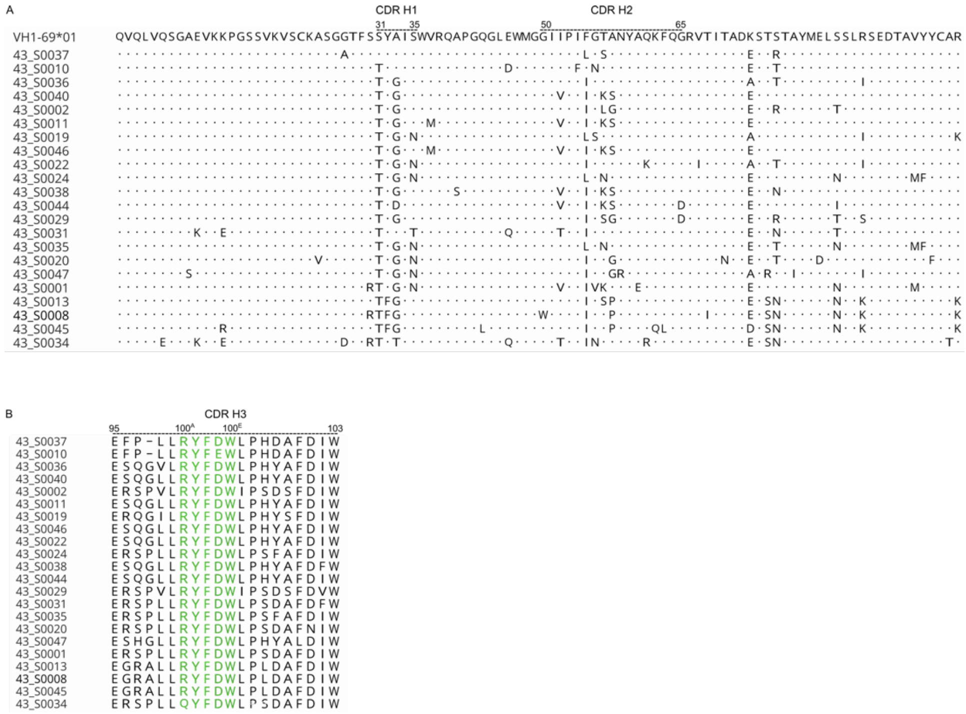

Figure S2. Heavy chain sequences of the IGHD3-9 mAbs, Related to Figure 2

(A) Somatic hypermutations in the mAbs isolated after the H7ssF-H1ssF-H1ssF immunization regimen. Amino acid sequence of variable domain encoded by human IGHV1-69*01 was used as a reference sequence using Kabat numbering.

(B) Amino acid sequence alignment of CDR H3 sequences of the mAbs in this study shown in Kabat numbering.

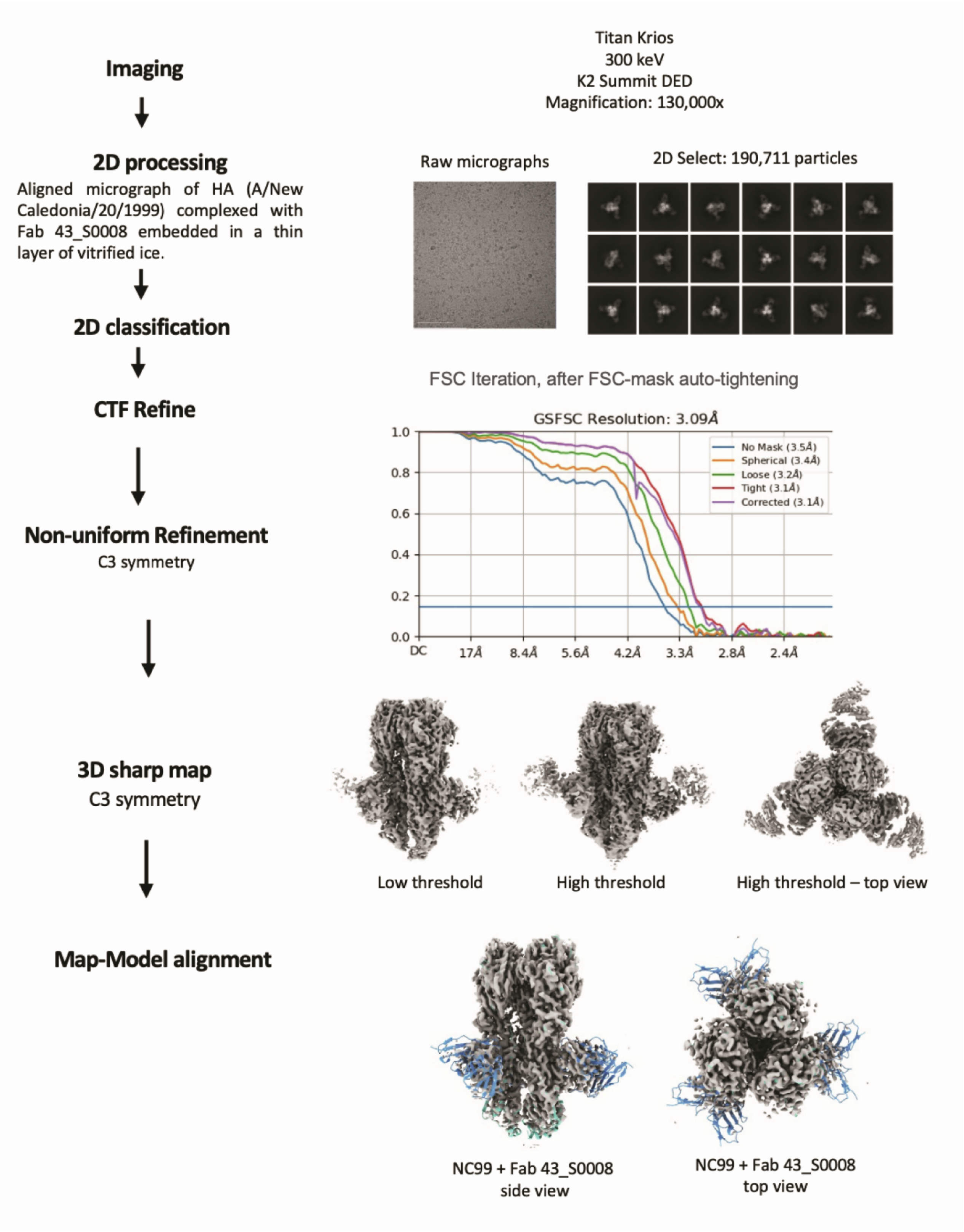

Figure S3. Cryo-EM processing summary, Related to Figure 3

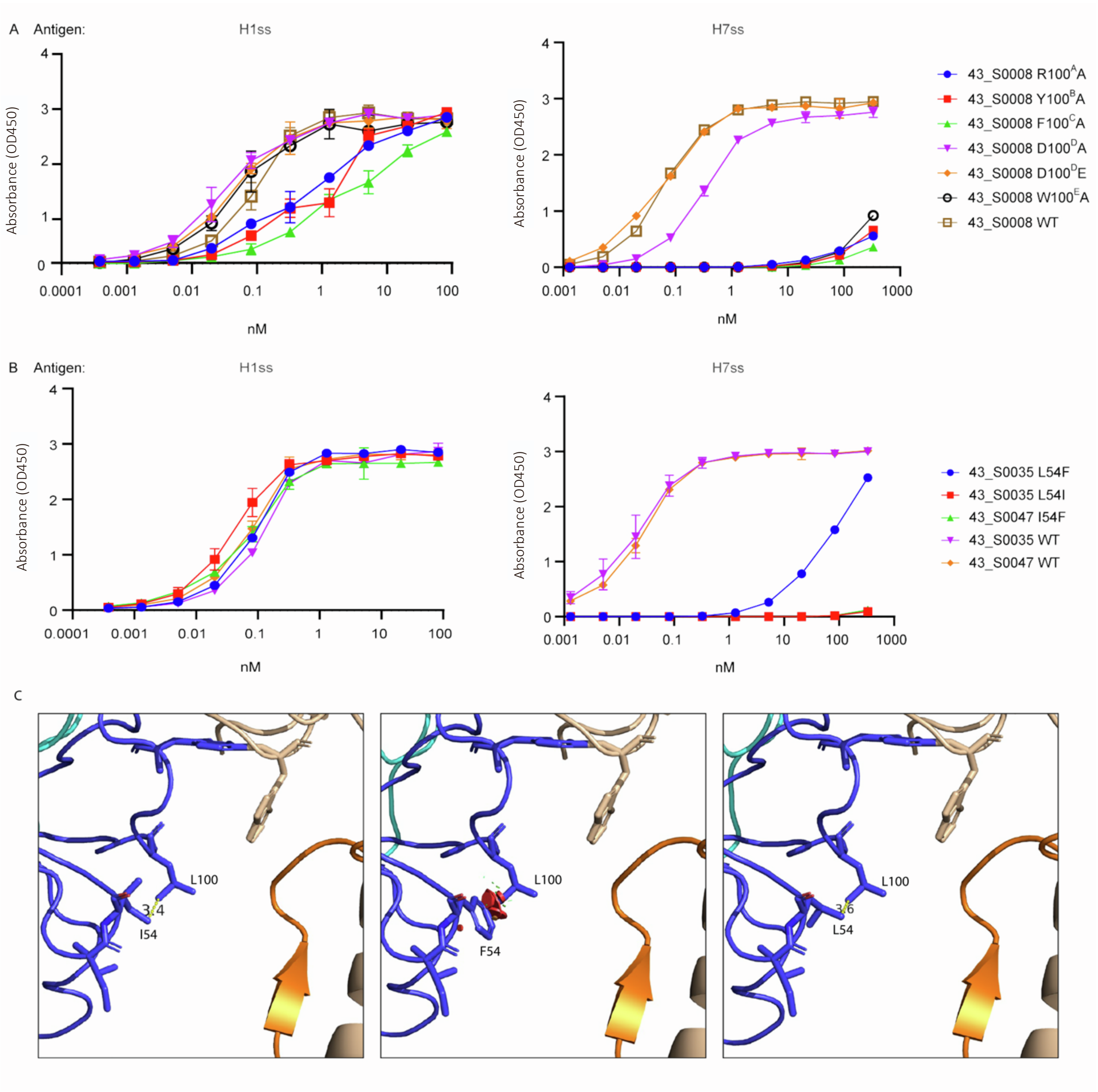

Figure S4. Binding profiles of select antibodies and their mutants to group 1 and group 2 HA antigens, Related to Figure 3

(A–B) Effect in IGHD3-9-derived mAb binding to group 1 and group 2 HAs. Substitution within the CDR H3 RYFDW motif (A), or CDR H2 IF motif (B).

(C) *In silico* mutagenesis of the CDR H2 residue 54 of 43_S0008. Observed structure of 43_S0008 (blue) with I54 in complex with HA (HA1, orange; HA2, beige) (left). *In silico* reversion of CDR H2 residue I54F (middle) or substitution I54L (right).
